# DeepGRP: engineering a software tool for predicting genomic repetitive elements using Recurrent Neural Networks with attention

**DOI:** 10.1186/s13015-021-00199-0

**Published:** 2021-08-23

**Authors:** Fabian Hausmann, Stefan Kurtz

**Affiliations:** 1 Institute of Medical Systems Biology, University Medical Center Hamburg-Eppendorf, Falkenried 94, 20251 Hamburg, Germany; 2 ZBH - Center for Bioinformatics, MIN-Fakultät, Universität Hamburg, Bundesstrasse 43, 20146 Hamburg, Germany

**Keywords:** Supervised Learning, Artificial Intelligence, Computational Predictions, Machine Learning Algorithms, Performance, Recurrent Neural Networks, Gated recurrent units, Attention, Repetitive elements, RepeatMasker, Satellite, DNA Sequences

## Abstract

**Background:**

Repetitive elements contribute a large part of eukaryotic genomes. For example, about 40 to 50% of human, mouse and rat genomes are repetitive. So identifying and classifying repeats is an important step in genome annotation. This annotation step is traditionally performed using alignment based methods, either in a *de novo* approach or by aligning the genome sequence to a species specific set of repetitive sequences. Recently, Li (Bioinformatics 35:4408–4410, 2019) developed a novel software tool dna-brnn to annotate repetitive sequences using a recurrent neural network trained on sample annotations of repetitive elements.

**Results:**

We have developed the methods of dna-brnn further and engineered a new software tool DeepGRP. This combines the basic concepts of Li (Bioinformatics 35:4408–4410, 2019) with current techniques developed for neural machine translation, the attention mechanism, for the task of nucleotide-level annotation of repetitive elements. An evaluation on the human genome shows a 20% improvement of the Matthews correlation coefficient for the predictions delivered by DeepGRP, when compared to dna-brnn. DeepGRP predicts two additional classes of repeats (compared to dna-brnn) and is able to transfer repeat annotations, using RepeatMasker-based training data to a different species (mouse). Additionally, we could show that DeepGRP predicts repeats annotated in the Dfam database, but not annotated by RepeatMasker. DeepGRP is highly scalable due to its implementation in the TensorFlow framework. For example, the GPU-accelerated version of DeepGRP is approx. 1.8 times faster than dna-brnn, approx. 8.6 times faster than RepeatMasker and over 100 times faster than HMMER searching for models of the Dfam database.

**Conclusions:**

By incorporating methods from neural machine translation, DeepGRP achieves a consistent improvement of the quality of the predictions compared to dna-brnn. Improved running times are obtained by employing TensorFlow as implementation framework and the use of GPUs. By incorporating two additional classes of repeats, DeepGRP provides more complete annotations, which were evaluated against three state-of-the-art tools for repeat annotation.

**Supplementary Information:**

The online version contains supplementary material available at 10.1186/s13015-021-00199-0.

## Introduction

As of May 2021, the *Genomes OnLine Database* (GOLD) [1] lists about 35 000 eukaryotic genome sequencing projects. Completion of eukaryotic genome sequencing projects is a difficult task, due to the large size of the genomes and the abundance of repeats in the genomes. For example, in the genomes of rat, mouse and human approximately 40 to 50% of the DNA consists of repeats [2] and these are divided into many different and partly not easily distinguishable classes.

The function of repetitive elements has been discussed for a long time [3] and only recently has the importance of repeats in cellular processes begun to open up [4]. Repetitive elements are important as binding regions for proteins, for example, involved in cellular replication [5] and they contain signals for transcription, chromatin assembly, nuclear localization [6] or influence expression of coding sequences [7]. Additionally, repetitive elements seem to have an impact on cell replication and genome structure evolution [8]. A recent overview of functions and the evolution of repetitive elements can be found in [9].

Due to these important roles of repeats, their annotation and classification in assembled eukaryotic genomes is an essential part of a genome project. In turn, a complete annotation of repeats allows to focus gene prediction on the remaining parts of the genome, like it is done in the Ensembl genome annotation system [10]. In this work we focus on four repeat classes for which we provide more details in the following paragraphs. While [11] mainly describes structural properties of two of the four repetitive element classes, we additionally highlight the biological importance and, if possible, function of specific repetitive elements.

Alphoid repeats or human alpha satellites are a human specific subclass of satellite DNA, which are long sequences of non-coding DNA appearing in tandems, i.e. the repeat instances follow each other without long gaps. They are mainly found in centromeric regions and usually have a length of around 171 bp [12]. Alphoid repeats are involved in the cell replication process by binding a protein for de novo centromere chromatin assembly [5]. In our RepeatMasker annotations of the reference assembly GRCh38 2.2% of the positions are annotated as alphoid repeats.

Human satellite type II and III (HSAT2,3) are another class of simple satellite DNA [13]. Repeats of this class have no consistent repeat unit reference sequence. In [11] HSAT2,3 repeats are characterized as diverse variations of the ATTCC motif. In [14] a slightly different characterization of HSAT2,3 repeats is used. According to [14] and references therein HSAT2,3 abundance varies between populations and cases are documented where instances of this repeat class cover at least ${1\times 10^{7}}\,{\hbox {bp}}$. [13] remarks that HSAT2,3 repeats only cover a small part ($\approx$ 1.5%) of a complete human genome of a single male donor. In our annotation, HSAT2,3 repeats only cover $\approx$ 0.08% of the GRCh38 assembly (cf. Section *Data sets*). It is known that HSAT2,3 repeats are involved in centromere maintenance, genome stability and cancer development [13], but their detailed function is poorly understood. The subclass of HSAT3 repeats play a role in cellular stress response, e.g. against genotoxic chemicals or oxidative stress [15]. HSAT2,3 repeats are human specific by definition. However, repetitive elements with a similar structural pattern are present in other eukaryotes [2] as well. The repeats of class HSAT2,3 are much shorter than the other repeat classes considered here and lack a consistent repeat unit reference [13]. So fewer positions on the DNA sequence can be utilized for prediction, which makes them harder to learn. Furthermore, the occurrence of repeats of the different repeat IDs of HSAT2,3 is very imbalanced [16] and therefore the reference annotation of the chromosomes probably does not represent all repeat IDs equally well.

Alu elements are non-autonomous retrotransposons, usually classified as short interspersed nuclear elements. Retrotransposons are sequence elements, able to change their location in the genome through RNA intermediates [17]. In this context, *non-autonomous* means, that they are not able to catalyze retrotransposition independently. Instead, for retrotransposition [18], they use LINE-1 repeats, explained below. In our annotation of GRCh38, Alu elements consist of approximately 300 bp and cover 9.7% of the base pairs. It has been shown that Alu elements are involved in gene expression regulation [17]. Furthermore, they are relevant in evolutionary biology [19], to, for example, narrow down geographical ancestry of primates [20]. They are used in forensics to determine evolutionary relationships [21]. Alu elements are specific to primates [22], but several short interspersed nuclear elements with similarity to Alu elements also exist in rodents [23].

Long interspersed nuclear elements type 1 (LINE-1, for short) are autonomous retrotransposons present in most mammalian genomes [24]. However, most of them lost their ability to retrotranspose [25]. LINE-1 repeats are sequences of approximately 6000 bp containing two open reading frames. They form a very diverse class of repetitive elements. In the RepeatMasker annotation of GRCh38 there are 194 different repeat IDs of LINE-1 repeats covering 14.6% of the base pairs. The protein encoded by the first open reading frame has RNA interaction and protein-protein binding capabilities. The protein encoded by the second open reading frame is involved in retrotransposition [25]. Furthermore, LINE-1 retrotransposition has an impact on human health, because retrotranspositions into important locations, e.g. genes, can be linked to inheritable disease, like Haemophilia A [26].

### Repeat annotation

Several tools for repeat annotation have been developed. The most widely known tool for this task is RepeatMasker [2], which follows a sequence alignment-based approach. It relies on a well-curated collection of repetitive sequences, called Repbase [27]. Annotation is performed by computing local alignments of the genomic sequences and the repetitive sequences from Repbase, using standard alignment methods. Due to the high quality of Repbase and the use of sensitive alignment methods, RepeatMasker provides the current gold standard for repeat annotation. However, Repbase is not commercially free and RepeatMasker is slow due to the time-consuming alignment process [11].

A more recent approach to repeat annotation is based on profile Hidden Markov Models (pHMMs). The open access database Dfam [28] provides a large collection of multiple sequence alignments of families of repetitive sequences and pHMMs derived from these. Sequences are annotated based on hits of the pHMMs in the sequences. The hits are computed using the HMMER software [29]. As Dfam provides a comprehensive collection of repetitive elements of high quality, is freely available and easy to use, it is likely to become the state of the art for repetitive element annotation in the near future [28].

Recently, Li [11] described an approach to annotate repeats using recurrent neural networks (RNNs), and showed that it works well for HSAT2,3 and Alphoid repeats. In contrast to conventional feed-forward networks, in an RNN the status of the hidden layer also depends on the input of some previous time steps [30]. This allows the RNN to develop a simple kind of memory and process sequences of variable length [31]. The most widely used RNNs are gated RNNs [32], like the long short-term memory (LSTM) [33] and the gated recurrent unit (GRU) [34]. Both, LSTMs and GRUs, are based on the idea of creating a path through time where the gradients neither explode nor vanish. This allows gated RNNs to accumulate information over long timescales. Additionally they have the capability to forget parts of their internal memory. GRUs are simplified versions of LSTMs, with less parameters to be trained. So LSTMs usually slightly outperform GRUs in terms of prediction performance [35]. However, GRUs are faster in training and prediction compared to LSTMs [36]. Besides their use in machine translation [35], polyphonic music modeling [37] and natural speech modeling [38], recurrent architectures are also applied to solve problems related to biological data, for example in the field of genomics [39]. Singh et al. [40] proposes a recurrent model for prediction of gene regulation based on a technique called attention. This technique was first employed for neural machine translation [41]. The attention mechanism scales the input of the next layer using the hidden state of the input layer, and thus allows the network to focus on important regions and sequence motifs during prediction [40].

### Maximum scoring segments algorithm

Since most sequence classification problems require the same classification category per segment, several algorithms for this task have been developed. A segment is here defined as an interval in a sequence of scores. The algorithm for finding all maximum scoring segments (MSS) by [42] is used in many different applications in bioinformatics. Li [11] extended this algorithm by incorporating a minimum score threshold and an *X*-drop parameter. Such techniques have a long tradition in sequence analysis and were, e.g., already used in Blast [43]. MSSs with a score smaller than the minimum score threshold are ignored. If the current score drops too much (as specified by the *X*-drop parameter) below the maximum score reached so far, all previous segments encountered in the extension are not considered when extending the next segments. So the *X*-drop parameter influences how many contiguous negative scores are tolerated in the extension process. A more detailed description of the MSS algorithm, including pseudocode and an example application, can be found in Additional file 1: Section 1.

### Contribution

We have developed the Deep Learning based method dna-brnn of Li [11] further. Our main contribution is the engineering and implementation of a new software tool DeepGRP. By engineering we mean the reimplementation and enhancement of the architecture of dna-brnn in the widely adopted machine learning framework TensorFlow. This approach has several advantages: The use of TensorFlow allows to profit from the regular improvements incorporated into TensorFlow by a large active community of developers. For example, it allowed us to seamlessly integrate the attention mechanism into the network architecture and makes it possible to exploit the high computational speed of GPUs. Additionally, it leads to a generalization of the approach of Li [11] and improves usability of the resulting software tool. dna-brnn has no dependencies on other software (except for a C-compiler), which makes it easy to install. On the other hand, the dependency of DeepGRP on TensorFlow is not a large hurdle given the fact that in May 2020 the number of downloads of TensorFlow reached 100 million [44].

In contrast to [11] we have systematically explored the latitude of the model by applying a well-tested hyperparameter optimization technique to determine seven hyperparameters used in DeepGRP. These and more details of the training are well documented to allow reproducing our results.

In addition to these technical contributions, we show that the RNN-based approach of DeepGRP can handle two additional classes of repeats, one of which Li [11] considered not accessible by this approach. Furthermore, we show that DeepGRP, trained on RepeatMasker annotations, delivers accurate predictions of repeats annotated by Dfam and not overlapping with RepeatMasker annotations.

## Methods and data sets

### Network architecture

Our new software tool DeepGRP uses a similar network architecture as dna-brnn but with an additional attention layer. The layer improves the ability of the network to learn motifs including gaps and stretching over long ranges of the DNA sequence.

The input of the neural network is the DNA sequence for which repeats are to be predicted. In a first step, initial and terminal stretches of Ns are eliminated. In the second step, the forward strand and the reverse complement strand of the remaining sequence are both one-hot encoded by $5$ integers per position, where the fifth integer is used for handling occurrences of Ns. The one-hot encoding is fed into a bidirectional GRU. While these bidirectional inputs are widely used for sequential tasks, like text translation [45], here the reverse complement strand is fed into the network to learn orientation-independent features. To let the network focus on short term relations and to have multiple predictions per sequence position, the DNA sequence is processed in a sliding window approach. The windows of length 342 are slid over the DNA sequence with a step size of 50. For each nucleotide in the window, the GRU-model predicts a probability with respect to all considered repetitive classes. As the windows are overlapping, the model produces multiple predictions for each position. These are maximized to obtain the final predictions (see next subsection). The length of the windows was determined by hyperparameter optimization and is fixed for training and evaluation. The step size for each window can be chosen at prediction time. This enables a trade off between running time and prediction accuracy.

To reduce the number of parameters to be trained and to allow the network to learn the features independently of the orientation of the sequence, the forward and the reverse GRU layer share weights. In [46] it was shown, that sharing of weights in such bidirectional model improves the model prediction performance and considerably reduces the number of learnable parameters. The output of the GRU layers is averaged and, together with the last hidden state, it is fed into the attention layer. The hidden state is also averaged over the forward and reverse layer.

**Fig. 1:**
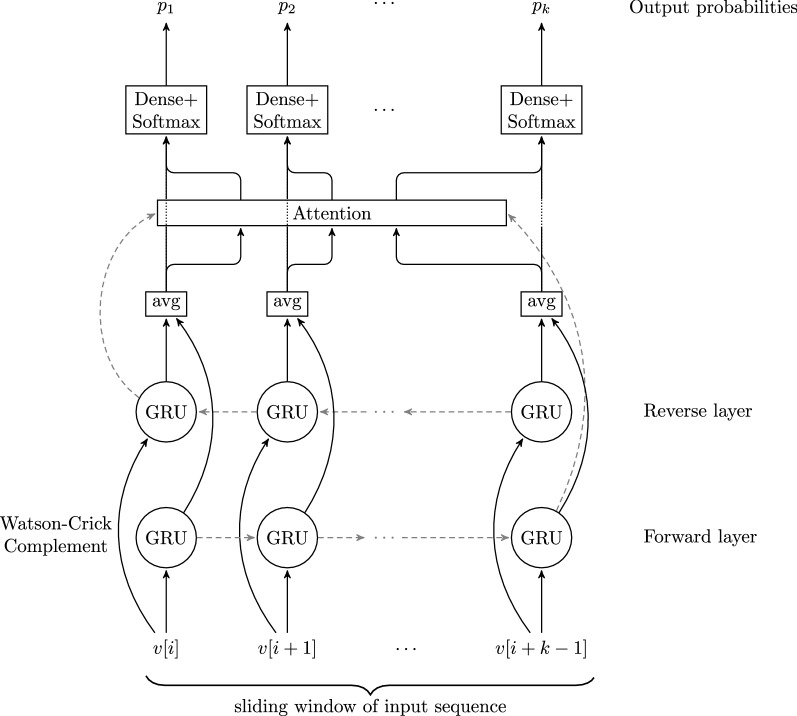
Sketch of the neural network architecture of DeepGRP. The architecture is similar to that of [11], but has an additional attention layer. The input flows along the black arrows, while the hidden states pass information along the dashed lines. The first input is the DNA sequence while the second input is the Watson-Crick complement of the sequence. Both sequences are one-hot encoded

The attention mechanism is implemented as proposed by [41]. It helps the network to utilize information from the complete sequence in a window, compressed as hidden state representation, at all sequence positions in that window. This improves the prediction of repetitive elements at the beginning of the genomic sequence and enhances the learning of more complex features. The output of the attention layer is fed, position by position, to a feed-forward network (Dense layer) with the Softmax activation function to obtain probabilities for each class of repetitive elements. The complete network is depicted in Fig. 1. One advantage of this approach is that all repetitive element classes can be handled at once and thus the number of repetitive elements has only a marginal influence on the running time.

### From probabilities to segments

Due to the window based approach with overlapping windows, for each nucleotide position and each repetitive class (including the no-repeat class) one obtains multiple probabilities of class memberships. For each position and each repetitive class we only keep the maximum of all such probabilities. This strategy of aggregating the information over different sliding windows leads to shorter running times and reduced space requirement, while still allowing for accurate predictions.

Let $C$ be the set of possible labels (each representing a repeat class) plus label 0 for class *no repeat*. Let $p_{i}^{c}$ be the probability derived by the model that position $i$, $1\le i\le n$ has label $c\in C$, where $n$ is the length of the sequence subject to the prediction of repeats. To derive predictions for segments of the input sequence, we first convert these probabilities to scores as follows: Calculate $q_{i} = \min \{\max \{p_{i}^{c}\mid c\in C\},0.99\}$ and determine $c_{i}\in C$ such that $q_{i}=\min \{p_{i}^{c_{i}},0.99\}$. Finally, convert $q_{i}$ to a score $s_{i}$ defined as follows [11]:

$$s_{i}= {\left\{ \begin{array}{ll} -10 \cdot \log \frac{q_{i}}{1-q_{i}} &{} \quad \text {if }c_{i}=0 \\ \log \frac{q_{i}}{1-q_{i}} &{} \quad \text {otherwise.} \end{array}\right. }$$

This scoring is a modified version of the ${\text {logit}}$-function, which is well known for its application in logistic regression [47]. The upper bounding to 0.99 prevents division by zero and $-10$ is multiplied for case $c_{i}=0$ to punish large extensions of segments. For this scoring approach we consider only the maximum probability per position over all repeat classes, as in the gold standard there are only few positions (i.e. 814 648 of 716 335 847 bp) for which the assignment to one of the four repeat classes considered here is not unique, see "Data sets" section. So applying the MSS algorithm to scores obtained from the probabilities for all classes of repetitive elements, would likely only lead to small improvements in accuracy, but mean a considerable increase of the computational effort. Therefore, the MSS algorithm is applied to the sequence $s_{1}$, $s_{2}$, ..., $s_{n}$ of scores obtained from the maximum probabilities. It delivers non-overlapping segments of continuous positions and for each position we have tracked the corresponding repeat class of maximum probability. *No repeat* positions inherit the repeat class which occurs most often in the segment. In this way, all positions of a segment are considered as part of an instance of one of the four considered repeat classes.

Afterwards, segments which contain more than one repetitive element class are split into several segments, each only containing one repetitive element class. Finally, the coordinates for all segments with a length greater than some user defined threshold (default 50, as also used in dna-brnn), are output.

### Implementation

The base network was implemented in Python (Version 3) using TensorFlow 2.1 [48] and is available as Python package deepgrp (https://github.com/fhausmann/deepgrp). Several time consuming steps are written in C. All C-codes including the the implementation of the MSS algorithm of [11] are integrated with DeepGRP via Cython.

### Data sets

Our evaluation is based on the reference assemblies GRCh19 and GRCh38 of the human genome (abbreviated as hg19 and hg38 in the following) and GRCm38 of the mouse genome (abbreviated as mm10). Details are given in Additional file 1: Table S2. To simplify notation, a specific chromosome $c$ of an assembly $a$ is denoted by $a/c$.

As the pre-annotated human genomes from the RepeatMasker website (http://www.repeatmasker.org/) are outdated, we computed the annotations of hg19 (all autosomal chromosomes) and hg38/chr1 ourselves, using Repbase 24.01 and RepeatMasker 4.1.0 (git-hash:41848148) [2], based on the cross_match [49] software computing the local alignments. The source code of cross_match was obtained from the author [50]. The annotations obtained in this way are considered as the gold standard for human. We found that 716 335 847 bp of the gold standard (i.e. 24.86% of hg19) are covered by a repeat of one of the four considered repeat classes. Of these 716 335 847 bp only 0.11% (814 648 bp) cannot uniquely be assigned to a repeat class.

As gold standard for mouse we used a pre-annotation of mm10/chr1 (cf. Additional file 1: Table S2). The main focus of using mouse data was to evaluate whether DeepGRP is able to make use of features learned from human sequences for annotating a different, but not too distant species which has specific repetitive elements. If not stated otherwise, the notation *gold standard* in singular means the gold standard for human.

From both gold standards we extracted all annotations of repeats of class HSAT2, Alphoid, Alu and LINE-1. A complete list of the repeat IDs of these classes (for human) can be found in Additional file 1: Table S3. Annotations for class HSAT3 were computed as described in [11]. As in [11] and [13], HSAT2 and HSAT3 were combined due to their similarity. All models were trained on hg19/chr11. For validation and early stopping hg19/chr20 was used. The true class labels were derived from the gold standard.

We selected hg19/chr11 for training for two reasons: On the one hand it was (besides other chromosomes) also used in [11] and on the other hand it is a medium sized chromosome ($\approx {4}{\%}$ of the bp. of hg19) and therefore represents a setting in which the size of the training data is small compared to the test data.

We selected hg19/chr20 for validation, since it is small ($\approx {2}{\%}$ of the bp. of hg19) allowing hyperparameter optimization in reasonable time. Early stopping was implemented in such a way that training is stopped, once the validation loss does not decrease in the last 10 epochs.

A Dfam-based annotation of repeats of the four repeat classes was obtained by extracting from Dfam release 3.3, November 2020, the related repetitive element classes from the pre-annotated hg38 genome. When determining the running time of HMMER, we used a subset of the Dfam HMM library (release 3.3) comprised of all classes of repetitive elements which were also present in the subset of the RepeatMasker annotations used in this study (cf. Additional file 1: Table S3). We applied dfamscan using HMMER (v3.2.1) [29], faithfully following the recipe of https://dfam.org/help/tools.

### Training details

While the basic network architecture we used is similar to the one proposed in [11], our training largely differs from the training of dna-brnn.

Training, retraining and evaluation were performed on Linux-based computers. The hyperparameter optimization was run on an Intel® Xeon® Gold 6142 CPU using 12 inter-operation and 12 intra-operation threads using Hyperopt with the *Tree of Parzen Estimators* algorithm [51]. Model performance is measured by the multi-class Matthews correlation coefficient, denoted by $MCC _{k}$ where $k$ is the number of classes [52]. $MCC _{k}$ is a single value characterizing a complete confusion table [52] even if the classes are very imbalanced [53]. In our application we have four different classes of repeats and the *no repeat* class, so $k$ is 5. We chose the model maximizing $MCC _{5}$. This model is termed best model.

The retraining of the best model and all evaluations, including training and evaluation of both tools, were performed on an Intel® Core® 7-5820K CPU using 10 threads. DeepGRP additionally utilized a GPU on a Nvidia Geforce GTX 960 graphics card using the XLA domain-specific compiler, see [54] for details. Training of DeepGRP required 6 to 15 minutes (median 11min) per model.

During the training, the composition of the batches is adjusted by a hyperparameter $r \in [0,1]$. A batch of size $n$ is constructed such that it contains at least $\lfloor n \cdot r\rfloor$ sequence windows, each of which has at least one position annotated as repetitive elements. This is further restricted such that from these $\lfloor n \cdot r\rfloor$ sequence windows for the set of all repetitive element classes $C$, $\left\lfloor \frac{n \cdot r}{|C|}\right\rfloor$ sequence windows per repetitive element class are present in one batch. Therefore, repeats which are less present in the training data are sampled more often during training to account for the imbalance of the occurrence of different repeat classes.

DeepGRP was compared to dna-brnn [11], which is implemented in C using its own neural network framework. Both programs use, as far as possible, the same hyperparameter values. dna-brnn does not provide an interface to perform hyperparameter tuning. Moreover, several parameters and flags of dna-brnn are hard coded or are ignored even if they have been specified on the command line. We concluded that hyperparameter tuning for dna-brnn would require modifications of its source code and the development of wrappers. As a consequence, we did not perform hyperparameter tuning for dna-brnn. For training and prediction the same hyperparameters were used.

dna-brnn uses a fixed number of epochs and a user defined random seed, whereas DeepGRP uses early stopping and a varying random seed. To the best of our knowledge, dna-brnn is not able to use validation data and the training cannot be resumed in previously saved states. So, it seems not possible to apply early stopping to prevent overfitting of parameters in dna-brnn. For DeepGRP we trained five models. Using different random seeds, we also trained five models for dna-brnn, in contrast to [11], in which only one model was trained.

## Results and discussion

In our initial experiments, DeepGRP and dna-brnn were evaluated based on the false positive rate (FPR) and the false negative rate (FNR). These rates describe easy to understand and relevant metrics. As in [11], these metrics refer to the base pair level: Let $G$ be the set of all positions in the considered sequence. Let $P_{c}$ be the set of positions in all predicted repeats of class $c$ and $P_{\lnot c} = G \setminus P_{c}$ be the set of positions predicted as another repeat class or as *no repeat*. Define $A_{c}$ and $A_{\lnot c}$ in an analogous way for annotated repeats from the gold standard. Then $(|P_{c}\setminus A_{c}|)/|A_{\lnot c}|$ is the false positive rate and $|A_{c}\setminus P_{c}|/|A_{c}|$ is the false negative rate for class $c$.

**Fig. 2:**
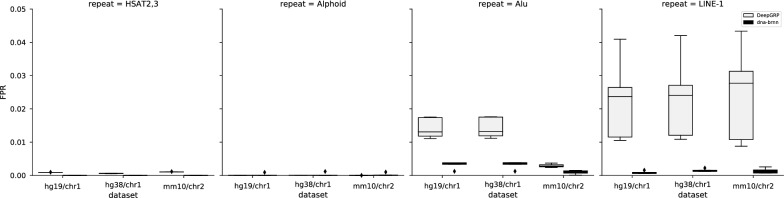
False positive rates (FPRs) for DeepGRP and dna-brnn, both for five models applied to hg19/chr1, hg38/chr1 and mm10/chr2. Training and validation of these models was performed on hg19/chr11 and hg19/chr20, respectively

**Fig. 3:**
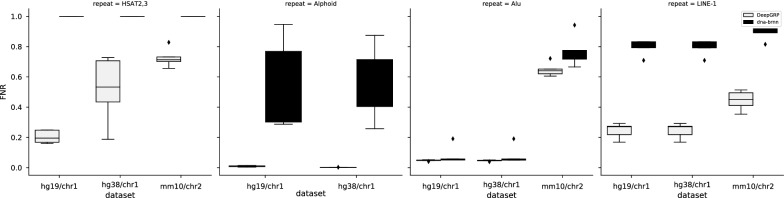
False negative rates (FNRs) for DeepGRP and dna-brnn, both for five models applied to hg19/chr1, hg38/chr1 and mm10/chr2. As Alphoid repeats are not annotated in mm10, the corresponding data is omitted

The FPRs and FNRs of the five models obtained for DeepGRP and for dna-brnn were determined for hg19/chr1, hg38/chr1 and mm10/chr2, which are the chromosomes of maximum sizes in the corresponding assemblies. The FPRs and FNRs of DeepGRP and dna-brnn are shown in Figs. 2 and 3, respectively. The FPRs of DeepGRP are very small (median $<0.03$) for all three data sets and all classes of repeats (cf. Fig. 2). The smallest FPRs are achieved for the classes HSAT2,3 and Alphoid (maximum FPR is 0.01). For the classes Alu and LINE-1, dna-brnn achieves slightly smaller FPRs than DeepGRP. As expected, the FNRs for hg19/chr1 and hg38/chr1 are smaller than for mm10/chr2, where the smallest FNRs are around 0.4. It is obvious that DeepGRP is able to predict repeats for all three chromosomes and all four considered classes. DeepGRP achieves smaller FNRs than dna-brnn. Except for Alu repeats, the FNRs of DeepGRP are much smaller than those of dna-brnn. According to the results of [11], dna-brnn is able to predict repeats of class HSAT2,3 for the assembly CHM1 of the human genome, achieving FNRs of $\approx$ 0.3%. But this result was achieved when considering only two classes of repeats (Alphoid and HSAT2,3). In our more challenging experiment with two additional classes (LINE-1 and Alu), dna-brnn achieved an FNR of 1.0 for HSAT2,3 repeats, i.e. it completely failed to predict repeats of this class. Another reason why our results concerning HSAT2,3 repeats differ from those reported in [11] may be due to the fact that for training of dna-brnn Li used additional annotated data. For three repeat classes (Alphoid, Alu, LINE-1) the FNRs for hg19/chr1 and hg38/chr1 are almost identical and very small. For hg38/chr1 and with respect to HSAT2,3, the median FNR is only slightly larger than 0.5, an issue considered below when we discuss misclassifications.

All models for dna-brnn and DeepGRP were trained on a single chromosome of hg19, which is much more similar to hg38 than to mm10. In particular, in mm10 one repeat class (Alphoid) is not present and some repeats (from class Alu and LINE-1) are specific for mm10 and do not occur in hg19/chr11 which served as training data. As a consequence, features of repeats learned from hg19/chr11 are much more likely to lead to correct predictions for hg38/chr1 than for mm10/chr2. Nevertheless, DeepGRP is able to correctly identify several repeats in mm10/chr2, for which it achieves considerably smaller FNRs than dna-brnn. This shows that DeepGRP is able to generalize across species.

**Fig. 4:**
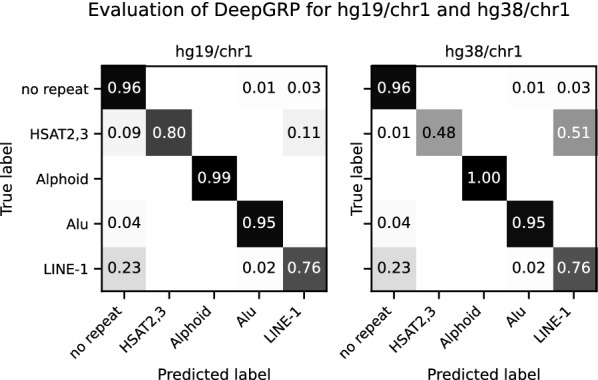
Confusion matrices for DeepGRP predicting repeats of four different classes for hg19/chr1 and hg38/chr1. The gold standard annotation was computed by RepeatMasker. Values are averaged over five models and absolute counts are divided by the true number (with respect to the gold standard) of annotated base pairs per repeat class. Values smaller than 0.01 are omitted. The models used for this evaluation are the same as for the other evaluations, i.e. trained on hg19/chr11

To study misclassifications of the models in more detail, we have created confusion matrices for the same five models applied to hg19/chr1 and hg38/chr1, see Fig. 4. Most misclassifications of repeat classes are related to LINE-1 repeats. In particular, DeepGRP classifies 23% of the LINE-1 repeats as *no repeat* (for hg19/chr1 and hg38/chr1) and 11% of HSAT2,3 repeats as LINE-1 repeats (for hg19/chr1). For hg38/chr1, 51% of the HSAT2,3 repeats are classified as LINE-1 repeats, i.e. DeepGRP is hardly able to correctly separate HSAT2,3 and LINE-1 repeats. To better understand the degradation in classification performance, we systematically determined sequence regions which are almost identical in both assemblies.

In particular, we computed colinear chains of approximate matches of hg19/chr1 and hg38/chr1. They make up 94.7% of hg19/chr1 and 92.6% of hg38/chr1 when not counting occurrences of Ns in the genomes. A detailed definition on how these chains are computed can be found in Additional file 1: Section 5. Considering only repeats in these common sequence regions, we obtain a highly improved accuracy for prediction of HSAT2,3 repeats, see Additional file 1: Figure S1. For the other three repeat classes there was virtually no differences in the prediction performance, but the number of base pairs in hg38/chr1 annotated as HSAT2,3 reduces to $\approx {6.6}{\%}$ (10 041 bp of 151 442 bp) according to gold standard annotation for hg38/chr1. This shows that regions annotated as HSAT2,3 are mainly in regions of hg38/chr1 differing from hg19/chr1. Also the total number of nucleotides annotated as HSAT2,3 (and Alphoid) differ considerably between regions common to hg19 and hg38, compared to regions unique to hg38, see Additional file 1: Figure S2. Due to this difference we cannot expect a good prediction performance for DeepGRP, especially for regions which are unique for hg38. Nevertheless, the results indicate that DeepGRP is able to learn sequences with no clear reference sequence without the need to be trained on exactly those sequences. RepeatMasker on hg19/chr1 compared to RepeatMasker on hg38/chr1 (see, Additional file 1: Figure S3) based on data from hgLiftOver [55] shows large overlaps of those annotations, for which the coordinates could be “lifted over” from hg38 to hg19. This provides additional evidence that DeepGRP performs well on regions common in different genome assemblies.

**Fig. 5:**
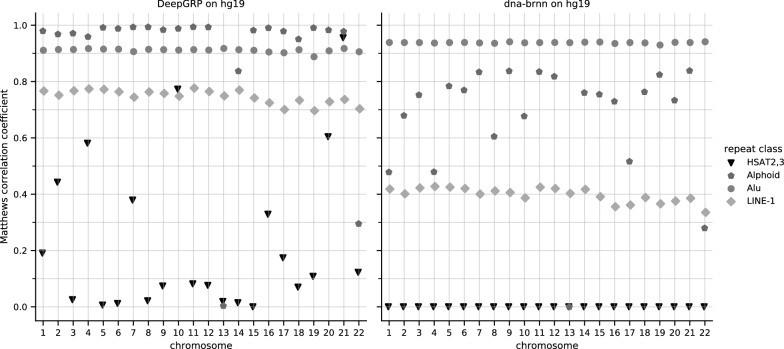
$MCC _{2}$ for DeepGRP and dna-brnn for all chromosomes of the human genome assembly hg19. For both tools five independently trained models were used. dna-brnn was trained with the same hyperparameter as DeepGRP for 50 epochs. All repeat classes where predicted with a single model, but the $MCC _{2}$-values where calculated in an one-vs-rest scheme, e.g. HSAT2,3 against not-HSAT2,3. The models used for this evaluation are the same as for the other evaluations, i.e. trained on hg19/chr11

While the results reported above were obtained for single chromosomes from the reference assemblies, we now report results obtained when applying the same five models to all autosomal chromosomes of hg19 (i.e. chr1, ..., chr22). The comparison is based on $MCC _{5}$-values (cf. Fig. 5). While DeepGRP achieves consistently high $MCC _{5}$-values for Alphoid, Alu and LINE-1, the $MCC _{5}$-values for HSAT2,3 varies. It seems more difficult for DeepGRP to learn elements of this class of repeats. This is likely due to the fact that repeats of class HSAT2,3 are much shorter and less conserved on the sequence level^1^ than repeats of the other three classes. The weaker performance of DeepGRP for HSAT2,3 and the complete failure of dna-brnn to predict HSAT2,3 repeats, as was already visible from the FNRs for selected chromosomes (see Fig. 3) is confirmed for all chromosomes of hg19. But at least, DeepGRP achieves $MCC _{5}$-values $\ge 0.8$ for three chromosomes of hg19. So one may conclude that DeepGRP is able to learn some repeats of class HSAT2,3 in a setting where only limited training data (i.e. 4% of hg19) is used.

For the prediction of Alu repeats dna-brnn slightly outperforms DeepGRP. This may be due to the fact, that during the training process, dna-brnn remains in a local minimum which allows to successfully predict Alu repeats, but delivers unreliable predictions of repeats of the other classes. In fact, for hg19/chr1 in almost all cases dna-brnn predicts the *no repeat* class or Alu repeats, see the confusion matrix of Additional file 1: Figure S4.

For Alphoid repeats DeepGRP clearly outperforms dna-brnn on all chromosomes and it shows much less variation between different models than dna-brnn (cf. Additional file 1: Figure S5). Interestingly, for Alphoid repeats on hg19/chr13 and hg19/chr22, the $MCC _{5}$-value drops for DeepGRP as well as for dna-brnn. As there is no difference in the number of segments or the number of annotated positions for Alphoid repeats for the two chromosomes compared to all other chromosomes, the reason for this behavior remains unclear.

For LINE-1 repeats DeepGRP achieves $MCC _{5}$-values (overall mean $\approx 0.747$) nearly twice as large as the $MCC _{5}$-values of dna-brnn (overall mean $\approx 0.397$). But the results for LINE-1 repeats are not as good as for Alphoid and Alu repeats. This is likely due to the fact that LINE-1 repeats have longer reference sequences (around 6000 bp [25]) than Alu and Alphoid repeats (<300 bp [13]). Moreover, LINE-1 repeats have a complex structure which is reflected by the division of LINE-1 elements into 194 different repeat IDs in Repbase. Nevertheless, LINE-1 repeats share a common structure and similar sequences. DeepGRP seems to be able to learn these much better than dna-brnn.

To evaluate whether DeepGRP is able to learn repeat annotations obtained by powerful models derived from sequence families, we compared its predictions to annotations of hg38 provided by the Dfam-database of transposable elements. While DeepGRP was trained on a gold standard computed by RepeatMasker, the confusion matrix of Additional file 1: Figure S6 shows that DeepGRP can predict repeats of class Alphoid, Alu and HSAT2,3 annotated in Dfam with an accuracy similar to the accuracy obtained when comparing with the gold standard. For LINE-1 repeats the accuracy in comparison to the Dfam annotation is lower than in the comparison to the gold standard. DeepGRP performs well in comparison to Dfam (cf. Additional file 1: Figure S7), although the overlap of the annotations of Dfam and RepeatMasker is only around 50% (cf. Additional file 1: Figure S3). Interestingly, DeepGRP can correctly predict repetitive elements annotated by Dfam, which are not present in the gold standard (Additional file 1: Figure S8). For example, DeepGRP correctly annotates 77% of Alphoid repeats, which are specific to Dfam.

Comparing only the boundaries of repeats (instead of all nucleotide positions of repetitive elements) to the gold standard delivered by RepeatMasker, shows a less convincing performance of DeepGRP. That is, only in rare cases, the boundary positions of repeats are precisely predicted. For example, for Alu repeats, maximum difference of boundaries of 50 bp. leads to a sensitivity of $\approx$ 60% and a specificity of $\approx$ 20%. The results for the other repeat classes are even worse, see Additional file 1: Figure S11 for details. We consider three main reasons for these results: The quality measures we apply hardly tolerate any misclassifications of single positions, see Additional file 1: Section 4. Thus a single false prediction has a much larger impact on the quality measures for repeat boundaries than on the quality measures based on nucleotide positions. Furthermore, boundaries of repetitive elements in the gold standard are ambiguous as they heavily depend on the number of allowed mismatches and indels when matching reference repeat sequences against a genome. Finally, in the training of DeepGRP all positions of repetitive elements are treated in the same way and no special emphases is laid on the boundary positions of the repeats, while RepeatMasker likely applies polishing techniques on the ends of “raw” repeats. So, additional enhancements with focus on polishing boundaries of repetitive elements and their integration into the current DeepGRP-model seems an interesting topic for further research. Of course, the relevance of these results on boundary predictions must be considered in the context of a concrete downstream analysis task for predicted repeats.

**Fig. 6:**
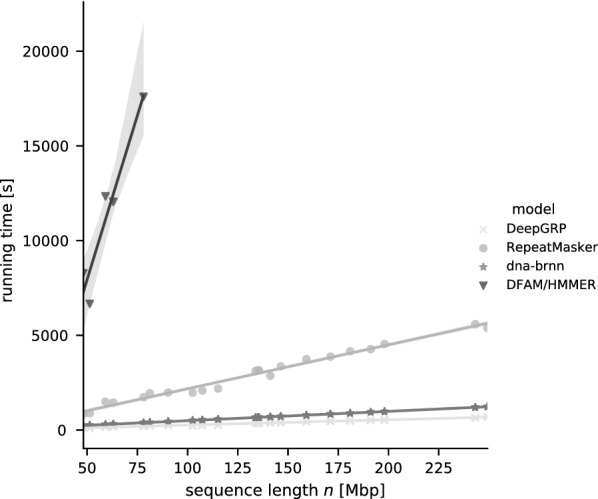
Prediction time of RepeatMasker, dna-brnn, DeepGRP and HMMER as a function of the sequence length in Mbp. For dna-brnn and DeepGRP five independently trained models and chromosomes from hg19 were used. dna-brnn was trained with the same hyperparameter as DeepGRP for 50 epochs with varying seed. The running time of dna-brnn and of DeepGRP shows almost no variance

One of the main goals when developing DeepGRP was to reduce the running time when annotating repeats. To prove that this goal was achieved, we measured the running time of the different software tools when annotating repeats of the four classes in hg19. Again, for dna-brnn and DeepGRP we used five trained models. Due to the long running time of HMMER [29] it was applied only to the five smallest autosomal chromosomes of hg19 (chr18, chr19, ..., chr22; total length 299 666 212 bp). For all three software tools, the running time for predicting repeats is linear (cf. Fig. 6). DeepGRP is approx. $8.6$ times faster than RepeatMasker, approx. $1.8$ times faster than dna-brnn (which runs on CPU only) and $>100$ times faster than HMMER. So DeepGRP, using a fairly old (6 years in 2021) GPU available on a standard graphics card outperforms all other methods.

## Conclusion

RepeatMasker [2] based on Repbase [27] and cross_match [49] for local alignment provides the current gold standard for annotation of repetitive elements in genomes. However, cross_match as well as Repbase are not commercially free and Repbase requires a paid license even for academic research. Dfam [28] and HMMER [29] are another powerful combination for annotating repetitive elements. While Dfam provides a comprehensive, well curated and free to use collection of HMMs for repeat identification and pre-annotated genomes for several genomes, the long running time of HMMER is a major hurdle when annotating repeats for complete genomes. dna-brnn and DeepGRP follow a new strategy to annotate repeats. The strategy is based on learning from already available repeat annotations and efficiently transferring this knowledge to new genome assemblies. Of course, this strategy is based on high quality repeat annotations (likely delivered by RepeatMasker), but it is independent of restrictions imposed by the license conditions of Repbase.

With the development of dna-brnn Li [11] showed how to implement the said strategy based on neural networks. Here we extend this idea further in several directions:

- By incorporating two new repetitive element classes, we provide more complete annotations.
- By employing TensorFlow as implementation framework for DeepGRP, we allow training and evaluation of the model to be executed on all widely used platforms including GPUs to reduce running times.
- By adding methods from neural machine translation, we achieve a consistent improvement of the quality of the predictions, compared to dna-brnn.

For the model with four classes of repeats, DeepGRP delivers considerably improved predictions ($MCC _{5}\approx 0.82 \pm 0.02$) compared to dna-brnn $MCC _{5}\approx 0.68 \pm 0.06$. For the two class model considered in [11], dna-brnn provides slightly better results ($MCC _{3}\approx 0.85$), but one should keep in mind that the simultaneous prediction of four classes of repeats is much more difficult. Our results obtained for relatively small amounts of training data (only 4% of the bp. of hg19) provide evidence that DeepGRP is able to generalize features learned from one genome assembly and transfer them to another assembly of the same species, but also with reduced precision to another related species.

Generally DeepGRP delivers conservative predictions, i.e. an annotation is rather missed than falsely predicted. This is an advantage in contexts where repeat masking is used for preprocessing sequences, before applying other tools to the unmasked part of the sequences for further annotation. In such a context a missed repeat would increase the number of base pairs to be annotated and thus lead to longer running times. A falsely predicted repeat would lead to masking regions possibly containing functional elements and thus have a negative effective of the sensitivity of the downstream annotation pipeline. A quantitative evaluation of the masking performance of DeepGRP can be found in Additional file 1: Figure S10.

DeepGRP is robust with respect to the training data. That is, when trained on different builds of the human genome, DeepGRP delivers the same prediction performance on all tested human genome assemblies, see Additional file 1: Figure S9. So, by training it directly on Dfam annotations could lead to an improved sensitivity of the prediction, in comparison to models that were trained only on RepeatMasker annotations. This could especially be useful in cases where high sensitivity is needed, but the running time is limited. Such an application could be subject for future research.

These properties and its improved running time makes DeepGRP a useful tool for annotating repetitive elements in eukaryotic genomes.

## Supplementary Information

**Additional file 1.** MSS algorithm and additional figures. Section 1: Detailed description of the maximum scoring segment algorithm and its implementation by [11]. Section 2: Additional tables. Section 3: Additional figures. Section 4: Sensitivity, specificity and the *δ*-parameter in the evaluation of repeat boundaries. Section 5: Detailed description of the comparison of hg19 and hg38.

## Data Availability

DeepGRP is available as Python package deepgrp (https://github.com/fhausmann/deepgrp) or on PyPI (https://pypi.org/project/deepgrp/). It is published under the Apache-2.0 License. Scripts to reproduce the results reported here can be found at https://github.com/fhausmann/deepgrp_reproducibility. DeepGRP is platform independent as long as the platform supports TensorFlow 2.1 or higher (up to TensorFlow 2.5). All genome assemblies used in the experiments are available at https://hgdownload.cse.ucsc.edu/goldenPath. Pre-annotated genomes are available at the RepeatMasker website (http://www.repeatmasker.org/genomes/) and the Dfam website (https://dfam.org/releases/Dfam_3.3/annotations). A detailed description of the used data sets can be found in Additional file 1: Table S2.

## Footnotes

1 This may be the reason why Dfam (release 3.3, November 2020) does not contain any repeats of class HSAT3 and relatively few of class HSAT2, see Additional file 1: Table S4.
