# Supplementary material for: DeepGRP: engineering a software tool for predicting genomic repetitive elements using Recurrent Neural Networks with attention
Source: Algorithms Mol Biol. 2021 Aug 23;16:20. doi: 10.1186/s13015-021-00199-0 (PMC8381506; doi:10.1186/s13015-021-00199-0)
Supplement: Supplementary file 1 — Additional file 1. MSS algorithm and additional figures. Section 1: Detailed description of the maximum scoring segment algorithm and its implementation by [11]. Section 2: Additional tables. Section 3: Additional figures. Section 4: Sensitivity, specificity and the δ-parameter in the evaluation of repeat boundaries. Section 5: Detailed description of the comparison of hg19 and hg38. [file 13015_2021_199_MOESM1_ESM.pdf]

DeepGRP: Engineering a Software Tool for Predicting Genomic Repetitive Elements Using Recurrent Neural Networks with Attention

# - Supplemental Material -

Fabian Hausmann and Stefan Kurtz

1 Detailed description of the Maximum scoring segment algorithm

Many classification problems of sequences require the same classification category per segment. Several algorithms for this task have been developed. However, in computational biology the algorithm for finding all maximal scoring segments (MSS) by [4] is widely used for this task [8, 2, 3, 9, 6]. A MSS is defined as follow:

Definition S1 (Maximum scoring segment)**.** *Let S be a sequence of n scores $S_1, S_2, \ldots, S_n$. Let $S_{a,b} = \sum_{i=a}^{b} S_i$ be the score of segment $[a, b]$ in S. $[a, b]$ is a maximum scoring segment in S if $S_{a',b'} < S_{a,b}$ for all a', b', $1 \leq a' \leq a$, $b \leq b' \leq n$ and $(a', b') \neq (a, b)$.*

An important property of MSSs is that they are non-overlapping (Theorem S1) and therefore have an unique solution for each position in the sequence of scores.

Theorem S1 (Non overlapping MSS)**.** *MSSs are non-overlapping.*

Proof. Let $[a, b]$ and $[a', b']$ be two different maximum scoring segments in S. Assume that they are overlapping. Without restricting generality assume that $a \leq a' \leq b$ and $a' \leq b \leq b'$. The score of the segments thus satisfies

$$S_{a,b} = S_{a,a'-1} + S_{a',b} \text{ and}$$
$$S_{a',b'} = S_{a',b} + S_{b+1,b'}$$

As $[a, b]$ is an MSS, $S_{a,b} > S_{a',b}$ which implies $S_{a,a'-1} > 0$. As $[a', b']$ is an MSS, $S_{a',b'} > S_{a',b}$ which implies $S_{b+1,a'} > 0$. The segment $[a, b']$ comprising both overlapping segments has a score

$$\begin{aligned} S_{a,b'} &= S_{a,b} + S_{a',b'} - S_{a',b} \\ &= S_{a,a'-1} + S_{a',b} + S_{a',b} + S_{b+1,b'} - S_{a',b} \\ &= S_{a,a'-1} + S_{a',b} + S_{b+1,b'} \end{aligned}$$

Substituting $S_{a',b} + S_{b+1,b'}$ by $S_{a',b'}$ implies $S_{a,b'} = S_{a,a'-1} + S_{a',b'}$. Substituting $S_{a,a'-1} + S_{a',b}$ by $S_{a,b}$ implies $S_{a,b'} = S_{a,b} + S_{b+1,b'}$. As $S_{a,a'-1} > 0$ and $S_{b+1,b'} > 0$, we conclude that $S_{a,b'} > S_{a,b}$ and $S_{a,b'} > S_{a',b'}$ which contradicts the initial assumption that $[a, b]$ and $[a', b']$ overlap. □

The algorithm is applied to a sequence of scores and can be described as follows [4]: The scores in S are processed from left to right and the cumulative score of scores in S is calculated. Additionally, an ordered list $I = (I_1, I_2, \ldots, I_m)$ of disjoint segments is maintained.

For each q, $1 \leq q \leq m$, the cumulative total score L_q of all scores up to but not including the leftmost score of I_q and the total score R_q up to and including the rightmost score of I_q is recorded. Formally:

$$L_q = \sum_{i=1}^{I_q.start-1} S_i$$

$$R_q = L_q + \sum_{i=I_q.start}^{I_q.end} S_i$$

Here $I_q.start$ is the start and $I_q.end$ the end position of a segment represented by I_q. $I.length$ is the number of segments stored in the list I, namely the list length.

Initially these lists, I, R and L, are empty. Scores ≤ 0 are skipped. A positive score at the first position or a positive score following a score ≤ 0 forms a new segment I_k of length one which is inserted into the previous lists using the following process:

1. Search for the maximum value of j, $1 \leq j \leq m$, satisfying $L_j < L_k$.
2. If there is no such j or $R_j \geq R_k$, then add I_k to the end of I.
3. Otherwise extend I_k to the leftmost position of segment I_j and update L_k accordingly. Delete segment $I_j, I_{j+1}, \ldots, I_m$ from I and continue with step 1 with $m = |I|$.

If the end of the input is reached, I is exactly the set of all MSS of S. A complete proof for the correctness of the algorithm can be found in [4].

The algorithm by [4], described above, is extended by [3] with a minimum score and a xdrop parameter [3]. If one of theses parameters is set to a negative values in the implementation by [3], the extension can be disabled. The minimum score parameter is used to filter the calculated MSSs. MSSs with a score smaller than the minimum score are dropped. The xdrop

parameter controls how large a segment of negative scores can be considered for extension. If the current score drops too much relative to the maximum score reached, all previous segments do not get considered for the extension of the next segments. idx and max are variables used for the xdrop application. Segments in I with an index smaller than idx are not considered for extension. idx is updated by comparing the current maximal value max with the current extended segment. The algorithm is shown in algorithm S1 and an example in table S1.

Table S1: Exemplary application of the MSS algorithm on a sequence of scores $S = [1, 1, -1, 1, 1, -4, 1, 1]$ based on the textual description. The algorithm terminates at the end of the sequence (Position 8) and found the MSSs $[1, 5]$ and $[7, 8]$ contained in I.

Position	Step	Condition	I_k	L_k	R_k	I	L	R
Initial						[ ]	[ ]	[ ]
1	Step 1	no j	[1,1]	0	1	[ ]	[ ]	[ ]
	Step 2					[1,1]	0	1
2	Step 1	$j = 1$	[2,2]	1	2	[1,1]	0	1
	Step 3		[1,2]	0	2	[ ]	[ ]	[ ]
	Step 1	no j	[1,2]	0	2	[ ]	[ ]	[ ]
	Step 2					[1,2]	0	2
3	skipped	$S_3 < 0$				[1,2]	0	2
4	Step 1	$j = 1$, but $R_1 \geq R_k$	[4,4]	1	2	[1,2]	0	2
	Step 2					[1,2], [4,4]	0, 1	2, 2
5	Step 1	$j = 2$	[5,5]	2	3	[1,2], [4,4]	0, 1	2, 2
	Step 3		[4,5]	1	3	[1,2]	0	2
	Step 1	$j = 1$	[4,5]	1	3	[1,2]	0	2
	Step 3		[1,5]	0	3	[ ]	[ ]	[ ]
	Step 1	no j	[1,5]	0	3	[ ]	[ ]	[ ]
	Step 2					[1,5]	0	3
6	skipped	$S_6 < 0$				[1,5]	0	3
7	Step 1	no j	[7,7]	-1	0	[1,5]	0	3
	Step 2					[1,5], [7,7]	0, -1	3, 0
8	Step 1	$j = 2$	[8,8]	0	1	[1,5], [7,7]	0, -1	3, 0
	Step 3		[7,8]	-1	1	[1,5]	0	3
	Step 1	no j	[7,8]	-1	1	[1,5]	0	3
	Step 2					[1,5], [7,8]	0, -1	3, 1

The algorithm, as shown in algorithm S1 and described textually, does not run in linear time, but several improvements regarding the implementation of the list structures can be done to accelerate the list searches. Some of them are already shown in algorithm S1 and described

below. The shown optimizations and others achieve linear running time in practice [4] and are implemented in [3]. The space requirements are clearly linear, since only maximal $\mathcal{O}(n)$ non-overlapping items in the list can exists, if n is the length of the sequence of scores. In practice these list are much shorter and further improvements can be made for the storing of the lists [4]. One of these improvements is shown in lines 3–7, where not each positive score separately, but a complete segment of positive scores is considered as initial $\Gamma\hat{=}I_k$. Another possible improvement for step 1 (lines 12–13) is instead of searching the full list I maintain for each $\Gamma\hat{=}I_k$ a pointer to previously discovered I_j and search this linked list of monotonically decreasing L_j values.

Algorithm S1 Maximum scoring segment algorithm. Based on [3].

```
findMSS(S, xdrop, min_sc)
I, R, L = [ ]                                              // Initially empty lists
ℓ = 0, max = −∞, idx = 1                                   // ℓ≙L_k in text
for (i = 1 to S.length)
    r = ℓ + S_i                                            // r≙R_k in text
    if (S_i > 0)
        for (z = i + 1 to S.length)
            if (S_z ≤ 0) break
            r = r + S_z
        Γ = (start = i, end = z)                           // Γ≙I_k in text
        max = MAX(max, r), stop = False
        while (True)
            for (j = I.length downto idx)                  // Step 1.
                if (L_j < ℓ) break
            if (j < idx or R_j ≥ r)                        // Step 2.
                if (j < idx) max = r
                I.append(Γ)
                R.append(r), L.append(ℓ)
                break
            else                                           // Step 3.
                ℓ = L_j
                Γ.start = I_j.start
                I = I_{1,j−1}
                L = L_{1,j−1}, R = R_{1,j−1}
            ℓ = r
    else
        if (ℓ + S_i + xdrop < max)
            ℓ = 0, max = −∞
            idx = I.length
        ℓ = ℓ + S_i
output = [ ]
for (z = 1 to I.length)
    if (R_z − L_z > min_sc) output.append(I_z)
return output
```

2 Additional Tables

Table S2: Data sets and gold standard annotations.

Data set name	Description	URL
hg19	DNA-sequences from human genome build hg19	https://hgdownload.cse.ucsc.edu/goldenPath/hg19/chromosomes/
hg38	DNA-sequences from human genome build hg38	https://hgdownload.cse.ucsc.edu/goldenPath/hg38/chromosomes/
mm10	DNA-sequences from mouse genome build mm10	https://hgdownload.cse.ucsc.edu/goldenPath/mm10/chromosomes/
RepeatMasker (hg19)	Custom computation, based on RepBase 24.01	https://www.girinst.org/repbase/reports/index.html
RepeatMasker (hg38)	Custom computation, based on RepBase 24.01	https://www.girinst.org/repbase/reports/index.html
RepeatMasker (mm10)	Precomputed annotations, available at RepeatMasker Website	http://www.repeatmasker.org/genomes/mm10/RepeatMasker-rm405-db20140131/mm10.fa.out.gz
Dfam (hg38)	Precomputed annotations for Dfam 3.3	https://dfam.org/releases/Dfam_3.3/annotations/hg38/hg38_dfam.nrph.hits.gz

Table S3: Repeat classes and corresponding repeat IDs from the gold standard for human. The list was created based on IDs from RepeatMasker preannotated genomes. IDs associated with descriptions matching the keyword LINE-1 were manually added.

Repeat class	Repbase	Dfam
Alphoid	ALR, ALR_, ALRa, ALRa_, ALRb, ALRb_	14, 15, 29
Alu	ALU, Alu, AluJb, AluJo, AluJr, AluJr4, AluSc, AluSc5, AluSc8, AluSg, AluSg4, AluSg7, AluSp, AluSq, AluSq10, AluSq2, AluSq4, AluSx, AluSx1, AluSx3, AluSx4, AluSz, AluSz6, AluY, AluYa5, AluYa8, AluYb8, AluYb9, AluYc, AluYc3, AluYd8, AluYe5, AluYe6, AluYf1, AluYg6, AluYh3, AluYh3a3, AluYh7, AluYh9, AluYi6, AluYi6_4d, AluYj4, AluYk11, AluYk12, AluYk2, AluYk3, AluYk4, AluYm1, FAM, FLAM_A, FLAM_C, FRAM	1145, 1154, 1169, 1174, 1197, 1240, 1316, 1317, 1318, 1320, 1321, 142, 143, 144, 147, 16, 2, 3, 34, 35, 36, 37, 38, 39, 40, 41, 42, 43, 44, 45, 46, 47, 48, 49, 50, 51, 52, 53, 54, 55, 56, 57, 58, 60, 63, 634, 64, 65, 66, 7, 73
HSAT2,3	HSATII	211
LINE-1	HAL1, HAL1M8, HAL1ME, HAL1b, L1, L1HS, L1M, L1M1, L1M1B_5, L1M1_5, L1M2, L1M2A1_5, L1M2A_5, L1M2B_5, L1M2C_5, L1M2_5, L1M2a, L1M2a1, L1M2b, L1M2c, L1M3, L1M3A_5, L1M3B_5, L1M3C_5, L1M3DE_5, L1M3D_5, L1M3a, L1M3b, L1M3c, L1M3d, L1M3de, L1M3e, L1M3f, L1M4, L1M4B, L1M4a1, L1M4a2, L1M4b, L1M4c, L1M5, L1M6, L1M6B, L1M6B_5end, L1M6_5end, L1M7, L1M7_5end, L1M8, L1MA1, L1MA10, L1MA2, L1MA3, L1MA4, L1MA4A, L1MA5, L1MA5A, L1MA6, L1MA7, L1MA8, L1MA9, L1MA9_5, L1MB1, L1MB2, L1MB3, L1MB3_5, L1MB4, L1MB4_5, L1MB5, L1MB6_5, L1MB7, L1MB8, L1MC, L1MC1, L1MC2, L1MC3, L1MC4, L1MC4B, L1MC4_5end, L1MC4a, L1MC5, L1MC5a, L1MCA_5, L1MCB_5, L1MCC_5, L1MCa, L1MCb, L1MCc, L1MD, L1MD1, L1MD1_5, L1MD2, L1MD3, L1MDA_5, L1MDB_5, L1MDa, L1MDb, L1ME1, L1ME2, L1ME2z, L1ME3, L1ME3A, L1ME3B, L1ME3C, L1ME3C_3end, L1ME3Cz, L1ME3D, L1ME3D_3end, L1ME3E, L1ME3E_3end, L1ME3F, L1ME3F_3end, L1ME3G, L1ME4, L1ME4A, L1ME4a, L1ME4b, L1ME4c, L1ME5, L1ME5_3end, L1MEA_5, L1MEB_5, L1MEC_5, L1MED_5, L1MEE_5, L1ME_ORF2, L1MEa, L1MEb, L1MEc, L1MEd, L1MEe_5end, L1MEf, L1MEf_5end, L1MEg, L1MEg1, L1MEg2, L1MEg_5end, L1MEh, L1MEi, L1MEj, L1P, L1P1, L1P2, L1P3, L1P3b, L1P4, L1P4a, L1P4a_5end, L1P4b, L1P4b_5end, L1P4c, L1P4c_5end, L1P4d, L1P4d_5end, L1P4e, L1P4e_5end, L1P5, L1PA10, L1PA11, L1PA12, L1PA12_5, L1PA13, L1PA13_5, L1PA14, L1PA14_5, L1PA15, L1PA15-16, L1PA16, L1PA16_5, L1PA17, L1PA17_5, L1PA2, L1PA3, L1PA4, L1PA5, L1PA6, L1PA7, L1PA7_5, L1PA8, L1PA8A, L1PB, L1PB1, L1PB2, L1PB2c, L1PB3, L1PB4, L1PBA1_5, L1PBA_5, L1PBB_5, L1PBa, L1PBa1, L1PBb, L1PREC1, L1PREC2, L1P_MA2, X9_LINE	10, 11, 1122, 154, 155, 156, 157, 199, 225, 226, 227, 228, 229, 230, 232, 233, 234, 236, 237, 238, 239, 240, 241, 242, 243, 244, 245, 246, 247, 248, 249, 250, 251, 252, 253, 254, 255, 256, 257, 258, 259, 260, 261, 262, 263, 264, 265, 266, 267, 268, 269, 270, 271, 272, 273, 274, 275, 276, 277, 278, 279, 280, 281, 282, 283, 284, 285, 286, 287, 288, 289, 290, 291, 292, 293, 294, 295, 296, 297, 298, 299, 300, 301, 302, 303, 304, 305, 306, 307, 308, 309, 310, 311, 312, 313, 314, 315, 316, 317, 318, 319, 320, 321, 322, 323, 324, 325, 326, 327, 328, 329, 330, 331, 332, 333, 334, 335, 336, 337, 338, 339, 340, 341, 342, 343, 344, 345, 346, 347, 348, 349, 350, 351, 352, 353, 354, 355, 356, 357, 358, 635, 771, 773, 774, 776, 777, 778, 8

Table S4: Number of elements (count), mean length, minimum length, and maximum length of the instances of the different repeat classes in the pre-annotated genome hg38 from Dfam (release 3.3, November 2020).

attribute	Alphoid	LINE-1	Alu	HSAT2
count	6.25e+04	1.25e+06	1.14e+06	9.18e+03
mean	1.65e+02	4.75e+02	2.62e+02	1.58e+02
min	2.50e+01	2.50e+01	2.10e+01	5.40e+01
max	2.44e+02	6.08e+03	5.56e+02	2.13e+02

3 Additional figures

The confusion matrices show averaged relative values, i.e. values are averaged over five models and absolute counts are divided by the true number (with respect to the gold standard) of annotated base pairs per repeat class. Values smaller than 0.01 are omitted. We used the same models as for the other evaluations, i.e. models trained on hg19/chr11.

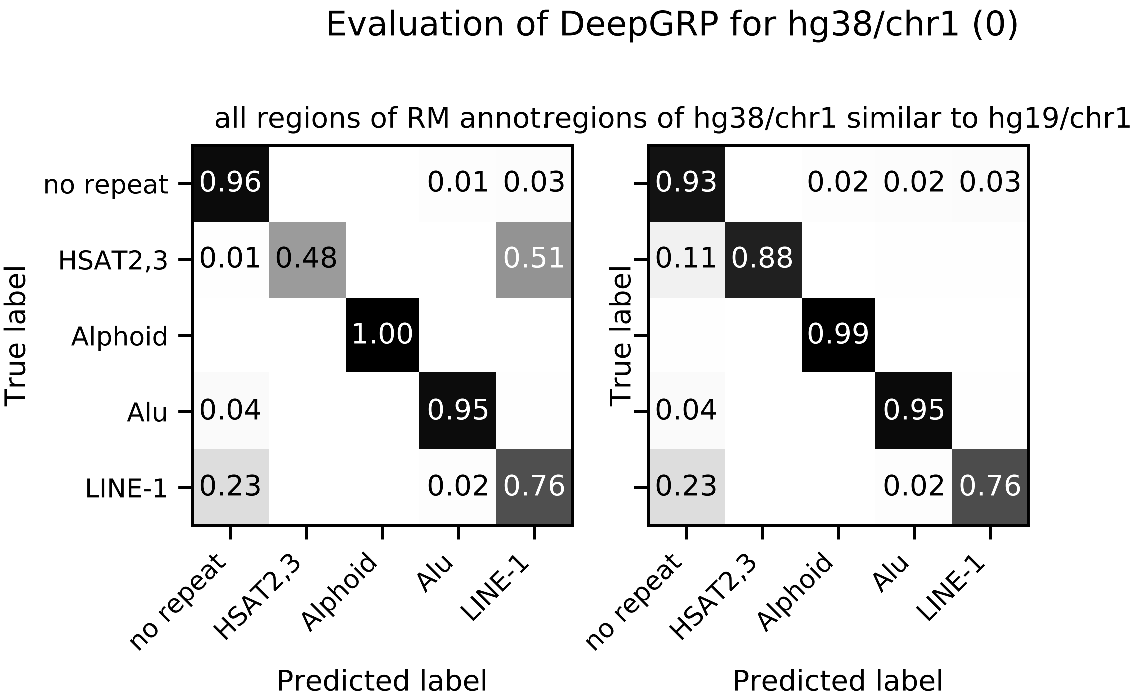

Figure S1: Confusion matrix comparing predictions by DeepGRP for hg38/chr1 with the gold standard; left: all of hg38/chr1; right: comparison restricted to sequences in chains of colinear approximate matches (length $\geq 50\,\mathrm{bp}$, mean length $542\,799\,\mathrm{bp}$, error rate $\leq 12.10\%$, overall error rate 0.0123%) in hg19/chr1 and hg38/chr1. These chains were computed as described in Section 5.

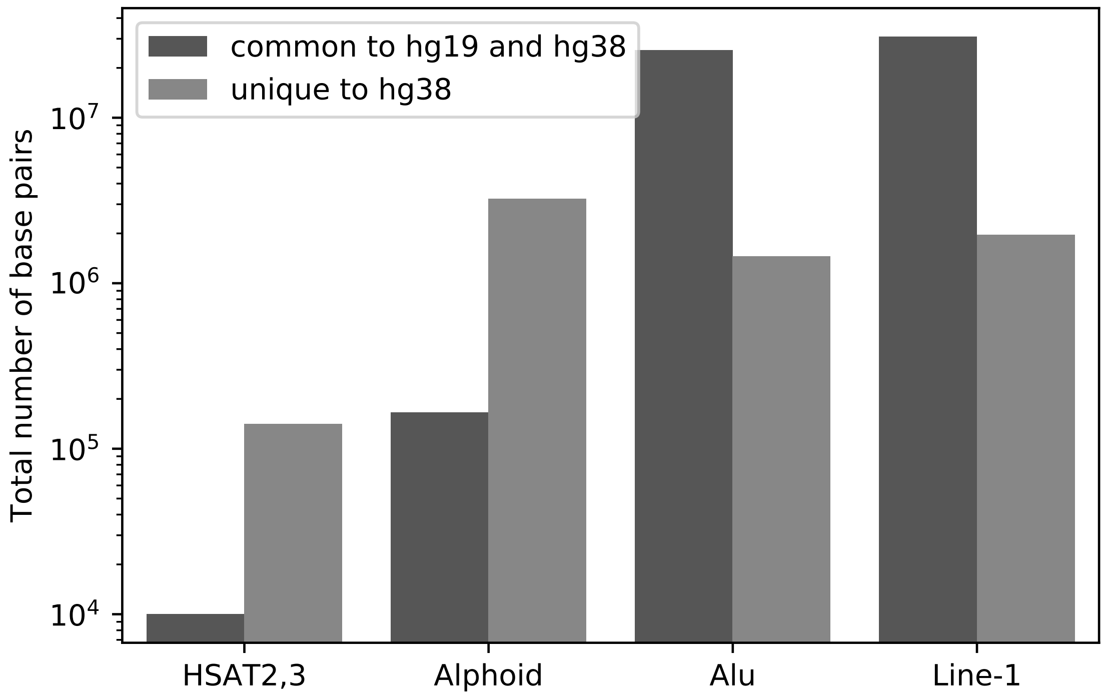

Figure S2: Total number of base pairs of chromosome 1 annotated as repetitive elements in regions which are common between hg19 and hg38 (sequences in chains of co-linear approximate matches, length $\geq 50\,\mathrm{bp}$, mean length $542\,799\,\mathrm{bp}$, error rate $\leq 12.10\%$, overall error rate 0.0123%) or sequences which are unique to hg38 (inverse of common regions).

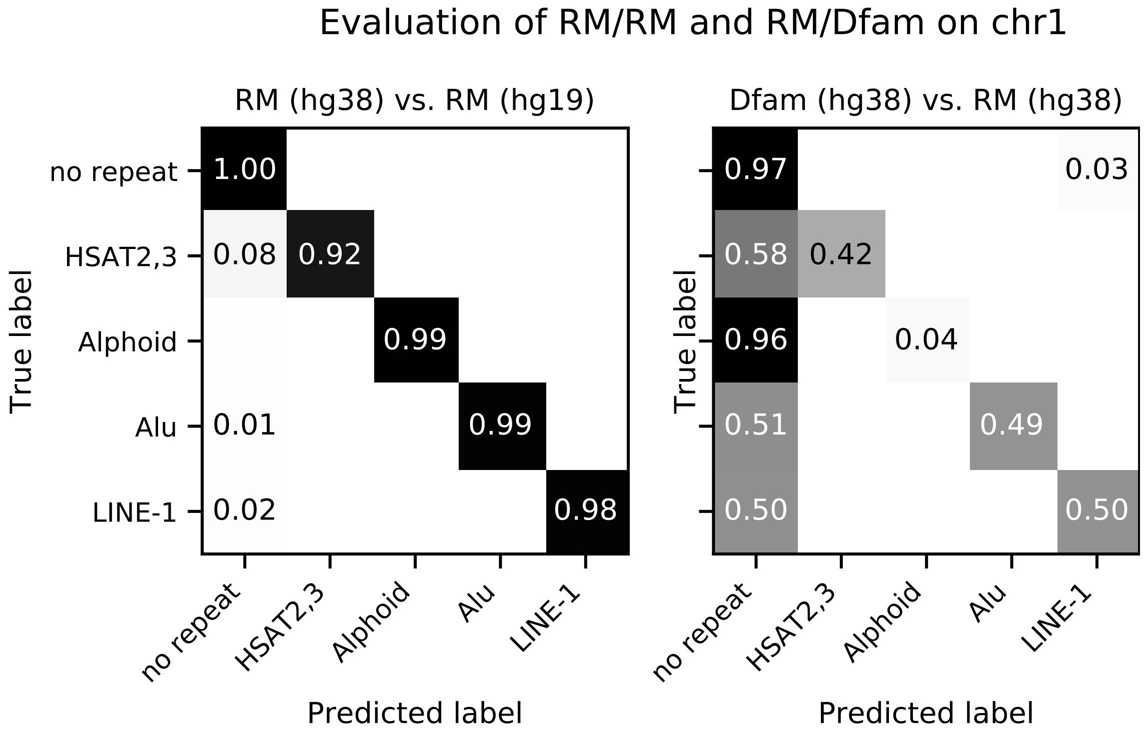

Figure S3: Confusion matrix resulting from the comparison of the predicted repeat annotations of RepeatMasker on hg38/chr1 compared to RepeatMasker on hg19/chr1 (left) and for Dfam [5] with RepeatMasker on hg38/chr1 (right). For the comparison of RepeatMasker on hg38 vs. hg19, hgLiftOver [7] was used to transfer the annotations from hg38 to hg19. For the comparison of RepeatMasker on hg38/chr1 to RepeatMasker on hg19/chr1 only annotations which could be transferred using hgLiftOver were considered.

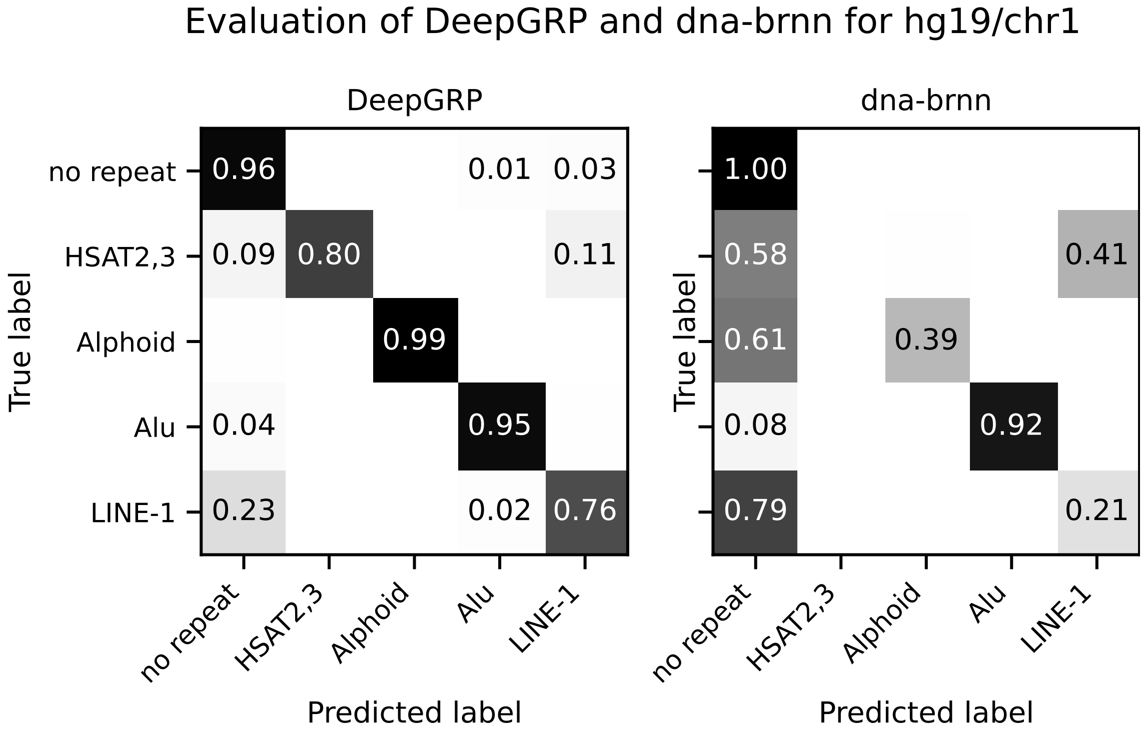

Figure S4: Confusion matrix resulting from the comparison of the predicted repeat annotations of DeepGRP for hg19/chr1 with the gold standard (left) and of dna-brnn for hg19/chr1 with the gold standard (right).

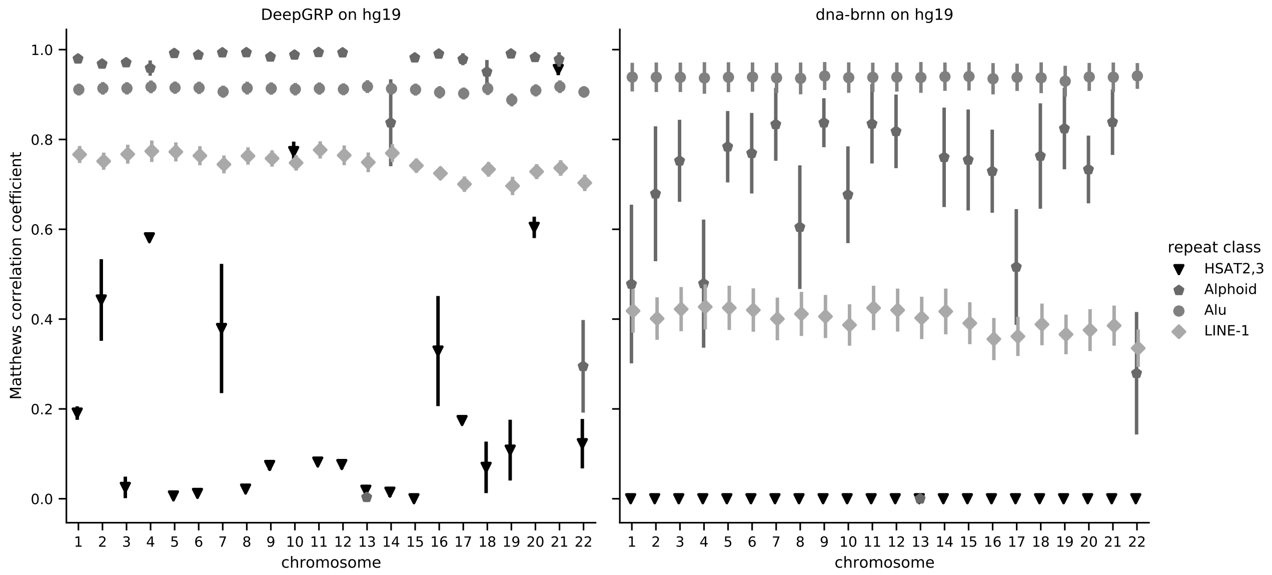

Figure S5: Mean MCC_2 shown with one standard deviation, for DeepGRP and dna-brnn for all autosomal chromosomes of the human genome assembly hg19. For both tools five independently trained models were used. dna-brnn was trained with the same hyperparameter as DeepGRP for 50 epochs. All repeat classes where predicted with a single model, but the MCC_2-values where calculated in an one-vs-rest scheme, e.g. HSAT2,3 against not-HSAT2,3. The models used for this evaluation are the same as for the other evaluations, i.e. trained on chromosome 11 of hg19.

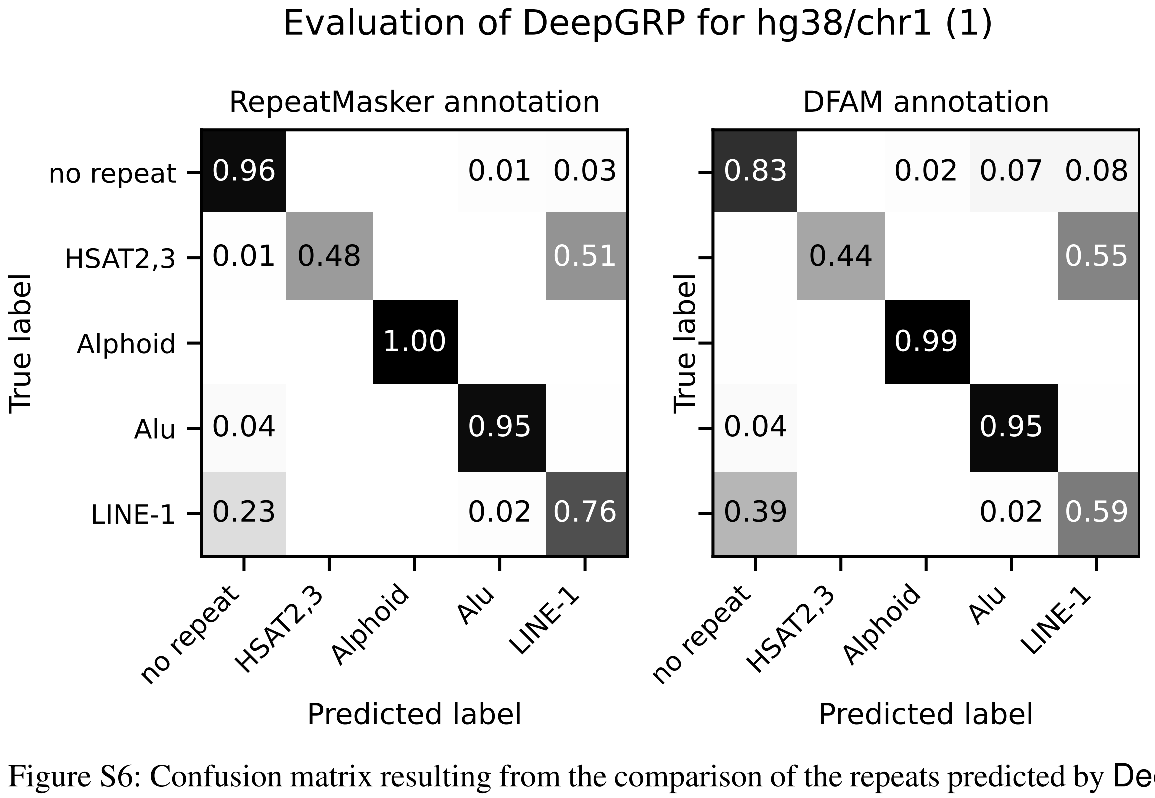

Figure S6: Confusion matrix resulting from the comparison of the repeats predicted by DeepGRP for hg38/chr1 with the gold standard (left) and with the annotation in Dfam [5] (right).

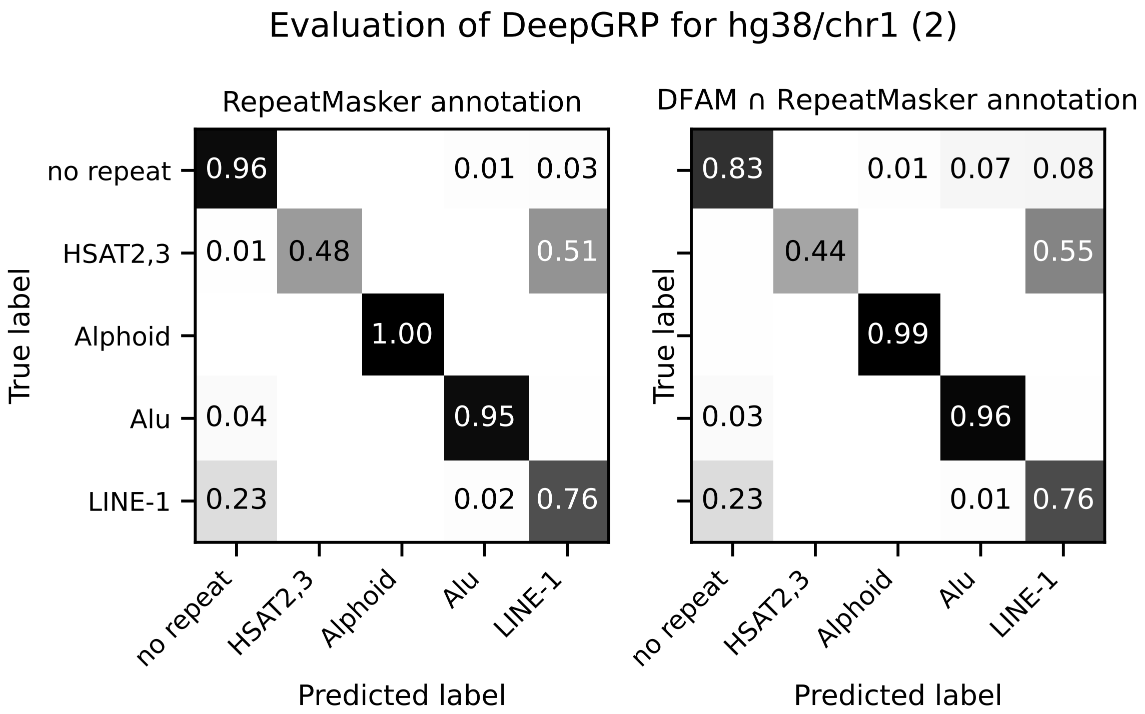

Figure S7: Confusion matrix resulting from the comparison of the repeats predicted by DeepGRP for hg38/chr1 with the gold standard (left) and with the intersection of the gold standard and the annotation in Dfam [5] (right).

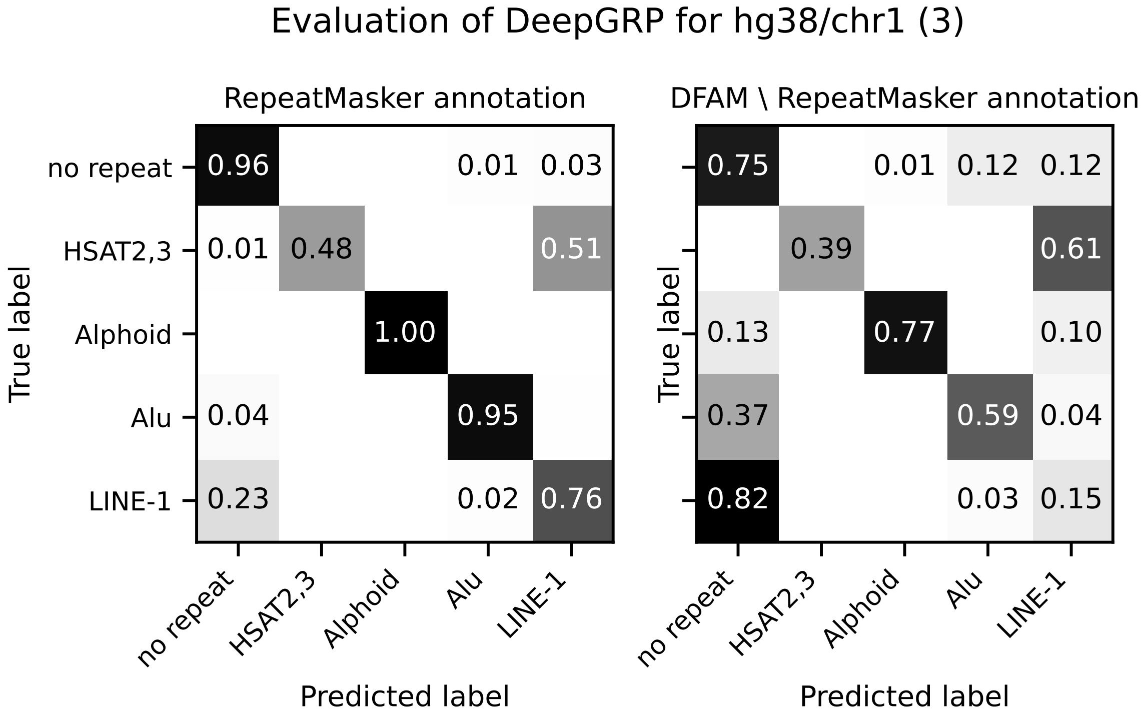

Figure S8: Confusion matrix resulting from the comparison of the repeats predicted by DeepGRP for hg38/chr1 with the gold standard (left) and with the annotation by Dfam [5] not in the gold standard (right).

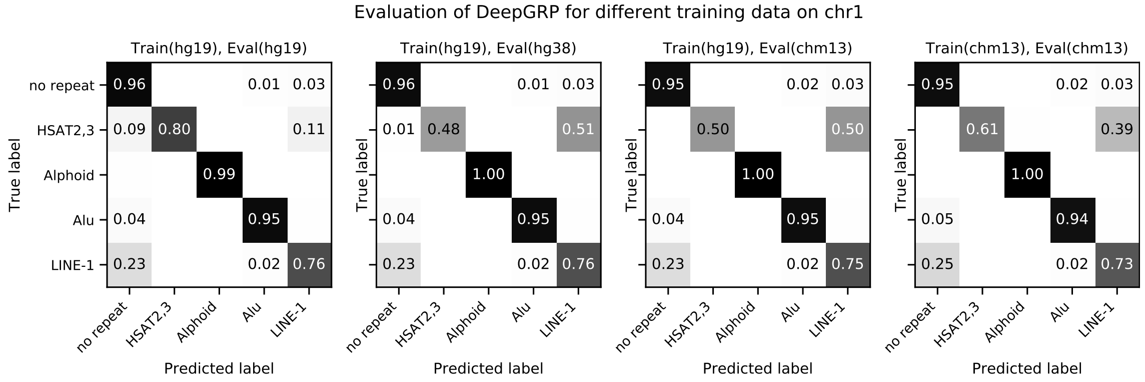

Figure S9: Confusion matrices resulting from the comparison of the predicted repeat annotations of DeepGRP with different training and evaluation data. For evaluation always chromosome 1 of the corresponding genome build was used.

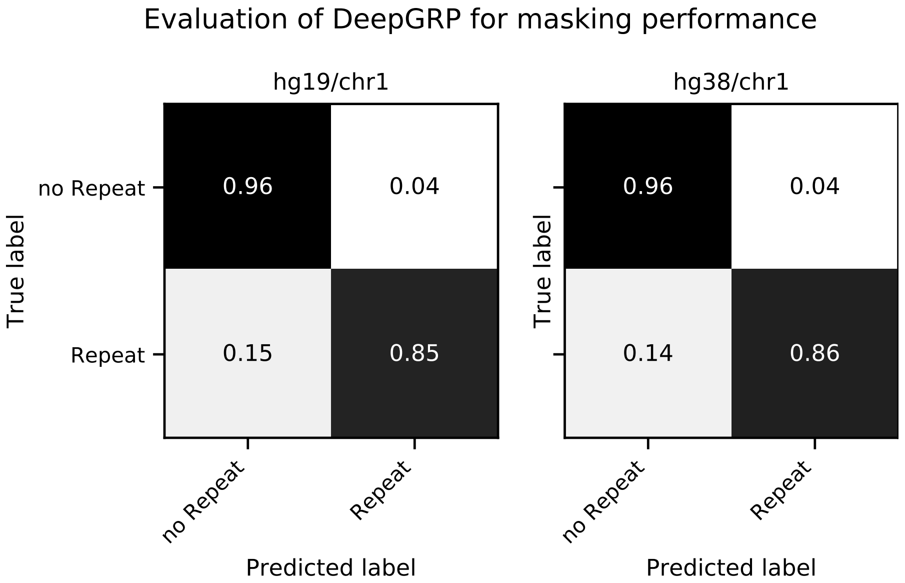

Figure S10: Confusion matrix resulting from the comparison of the repeats predicted by DeepGRP with the gold standard for hg19/chr1 (left) and hg38/chr1 (right), ignoring which specific repeat class was annotated. That is, for every position it is only considered if this is in a substring annotated as any repeat or as no repeat. Note that DeepGRP was still trained to predict the four considered repeat classes, which were only considered as a single 'any repeat' class for evaluation.

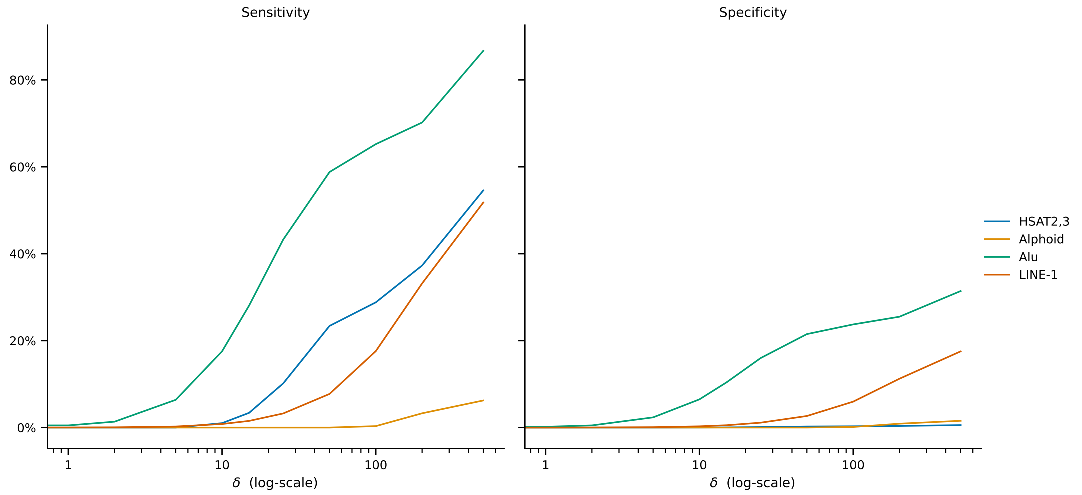

Figure S11: Sensitivity and specificity of predictions of DeepGRP for hg19/chr1, considering only the boundaries of repeats, as a function of the tolerance value $\delta \in \{0, 1, 2, 5, 10, 15, 25, 50, 100, 200, 500\}$, see Section 4 for details. As gold standard we used the annotation of RepeatMasker. The results are shown separately for each of the four repeat classes. A line represents the mean of the predictions of five models and the area around a line represents values within one standard deviation of the mean. We use the log-scale for the X-axis. The evaluation was performed by the program `gt eval` of the GenomeTools software package [1] using options `-ltr` and `-ltrdelta` δ.

4 Sensitivity, specificity and the δ-parameter in the evaluation of repeat boundaries

The parameter δ, see Figure S11 specifies the maximum difference allowed between the position of a boundary of overlapping repeats of the same type from the gold standard and the prediction to qualify as correct boundary. For a given value of δ, the sensitivity is the percentage of repeats of the gold standard whose boundary is correct in the prediction. The specificity is the percentage of repeats of the prediction whose boundary is correct in the gold standard. These quality measures do not tolerate any misclassifications with distance larger than δ from the boundaries of repeats of the gold standard. Consider, for example, an interval $[\ell \dots r]$ representing a repetitive element of class c that is correctly predicted for all positions except for a single position i, $\ell \leq i \leq r$, which is assigned to a class different from c. If the distance of position i to ℓ and r is larger than δ, this predicted repetitive element contributes 0 to the sensitivity and specificity measure. Thus, for constant δ, the larger the repeats, the more unlikely it is to achieve reasonable sensitivity and specificity values.

5 Identifying chains of colinear exact and of approximate matches in pairs of chromosomes of hg19 and hg38

To evaluate the difference between the training and test data, chromosomes from hg19 and hg38 where compared on a base-pair level. Since the chromosomes are to huge to compare using greedy alignment methods, the following approach was used:

1. Remove from the sequences the maximum prefix and suffix consisting of N's only.
2. Construct an enhanced suffix array for the concatenation of each chromosome from hg19 and hg38 using `gt suffixerator` from the GenomeTools [1].
3. Compute all maximal exact matches of length $\geq \ell$, where one instance of the match occurs in sequence of hg19 and the other in hg38. This is done by `gt repfind` from the GenomeTools [1].
4. Compute global co-linear chains of maximum score of all matches. This is done by `gt chain2dim` from the GenomeTools [1].
5. For chains compute the positions of the pairs of substrings before the first chained match, between two chained matches and after the last chained match. So all pairs of regions between the chained matches are determined.
6. For each pair of gaps, extract the corresponding pair of sequences from hg19 and hg38 and try to align them globally using a greedy alignment method with sensitive parameter settings. We either obtain an upper bound of the unit edit distance of the pair of sequences of the gap or an undefined value in case the alignment method was not able to compute such an upper bound.
7. Merge adjacent gaps (with defined upper bounds) and matches to obtain supermatches. A supermatch thus consists of a maximal sequence of closed gaps including the matches in between two consecutive gaps and the match before the first gap and after the last gap. The sum of the distance values gives an upper bound on the unit edit distance of the sequences of the supermatch. Note that the supermatches form a co-linear chain of non-overlapping approximate matches with no guaranteed upper bound on the error rate. In our application we see error rates of up to $50\,\%$. Two supermatches are separated by gaps for which the edit distance is too large to be computed by the greedy alignment methods.
8. Align the entire sequences of each supermatch to compute the unit edit distance, using a greedy alignment method. With very few exceptions, the long supermatches of more than $10\,000\,\mathrm{bp}$ have a very low error rate while the supermatches with higher error rates are shorter. So the alignment can be computed very quickly.

References

[1] G. Gremme, S. Steinbiss, and S. Kurtz. GenomeTools: a comprehensive software library for efficient processing of structured genome annotations. *IEEE/ACM transactions on computational biology and bioinformatics*, 10(3):645–656, 2013.

[2] S. Karlin and S. F. Altschul. Applications and statistics for multiple high-scoring segments in molecular sequences. *Proceedings of the National Academy of Sciences*, 90(12):5873–5877, 1993.

[3] H. Li. Identifying centromeric satellites with dna-brnn. *Bioinformatics*, 35(21):4408–4410, 04 2019.

[4] W. L. Ruzzo and M. Tompa. A Linear Time Algorithm for Finding All Maximal Scoring Subsequences. In *Proceedings of the Seventh International Conference on Intelligent Systems for Molecular Biology*, pages 234–241, Palo Alto, California, USA, 1999. AAAI Press.

[5] J. Storer, R. Hubley, J. Rosen, T. J. Wheeler, and A. F. Smit. The Dfam community resource of transposable element families, sequence models, and genome annotations. *Mobile DNA*, 12(1):1–14, 2021.

[6] D. Sun and V. Y. Soojin. Impacts of chromatin states and long-range genomic segments on aging and DNA methylation. *PLoS One*, 10(6):e0128517, 2015.

[7] UCSC Genome Browser Development Team. Lift genome annotations, 2021.

[8] J. Wang, V. V. Lunyak, and I. K. Jordan. Genome-wide prediction and analysis of human chromatin boundary elements. *Nucleic Acids Research*, 40(2):511–529, 2011.

[9] J. Wang, V. V. Lunyak, and I. K. Jordan. BroadPeak: a novel algorithm for identifying broad peaks in diffuse ChIP-seq datasets. *Bioinformatics*, 29(4):492–493, 2013.
